# Clinical Features of Myasthenia Gravis With Antibodies to MuSK Based on Age at Onset: A Multicenter Retrospective Study in China

**DOI:** 10.3389/fneur.2022.879261

**Published:** 2022-04-08

**Authors:** Yufan Zhou, Jialin Chen, Zunbo Li, Song Tan, Chong Yan, Sushan Luo, Lei Zhou, Jie Song, Xiao Huan, Ying Wang, Chongbo Zhao, Wenshuang Zeng, Jianying Xi

**Affiliations:** ^1^Department of Neurology, Huashan Hospital, Fudan University, Shanghai, China; ^2^Huashan Rare Disease Center, Huashan Hospital, Fudan University, Shanghai, China; ^3^National Center for Neurological Diseases, Shanghai, China; ^4^Department of Neurology, Fujian Medical University Union Hospital, Fuzhou, China; ^5^Department of Neurology, Xi'an Gaoxin Hospital, Xi'an, China; ^6^Department of Neurology, Sichuan Provincial People's Hospital, University of Electronic Science and Technology of China, Chengdu, China; ^7^Chinese Academy of Sciences Sichuan Translational Medicine Research Hospital, Chengdu, China; ^8^Department of Pharmacy, Huashan Hospital, Fudan University, Shanghai, China; ^9^Department of Neurology, The University of Hong Kong-Shenzhen Hospital, Shenzhen, China

**Keywords:** myasthenia gravis, muscle-specific tyrosine kinase, clinical features, weakness distribution, age at onset

## Abstract

**Introduction:**

Antibodies to MuSK identify a rare subtype of myasthenia gravis (MuSK-MG). In western countries, the onset age of MuSK-MG peaks in the late 30's while it is unknown in Chinese population.

**Methods:**

In this retrospective multicenter study, we screened 69 MuSK-MG patients from 2042 MG patients in five tertiary referral centers in China from October 2016 to October 2021 and summarized the clinical features and treatment outcomes. Then we subgrouped the patients into early-onset (<50 years old), late-onset (50–64 years old), and very-late-onset (≥65 years old) MG and compared the differences in weakness distribution, disease progression and treatment outcomes among three subgroups.

**Results:**

The patients with MuSK-MG were female-dominant (55/69) and their mean age at onset was 44.70 ± 15.84 years old, with a broad range of 17–81 years old. At disease onset, 29/69 patients were classified as MGFA Type IIb and the frequency of bulbar and extraocular involvement was 53.6 and 69.6%, respectively. There was no difference in weakness distribution. Compared with early-onset MuSK-MG, very-late-onset patients had a higher proportion of limb muscle involvement (12/15 vs.16/40, *p* = 0.022) 3 months after onset. Six months after onset, more patients with bulbar (14/15 vs. 26/39, *p* = 0.044) and respiratory involvement (6/15 vs. 0/13, *p* = 0.013) were seen in very-late-onset than in late-onset subgroup. The very-late-onset subgroup had the highest frequency of limb weakness (86.7%, *p* < 0.001). One year after onset, very-late-onset patients demonstrated a higher frequency of respiratory involvement than early-onset patients (4/12 vs. 2/35, *p* = 0.036). 39/64 patients reached MSE. Among 46 patients who received rituximab, very-late-onset patients started earlier than late-onset patients [6 (5.5–7.5) vs. 18 (12–65) months, *p* = 0.039], but no difference in the time and rate to achieving MSE was identified.

**Conclusion:**

MuSK-MG patients usually manifested as acute onset and predominant bulbar and respiratory involvement with female dominance. Very-late-onset patients displayed an early involvement of limb, bulbar and respiratory muscles in the disease course, which might prompt their earlier use of rituximab. The majority MuSK-MG patients can benefit from rituximab treatment regardless of age at onset.

## Introduction

Myasthenia gravis (MG) is an autoimmune neuromuscular disorder characterized by circulating autoantibodies against functionally important components of the postsynaptic membrane, including acetylcholine receptor (AChR) and muscle-specific tyrosine kinase (MuSK). MuSK is essentially a neuromuscular junction protein, which is closely related to the assembly of AChR. MG with antibodies against MuSK (MuSK-MG) is a rare subtype and it is found that only 0–6% in patients with MG (1–4). MuSK-MG is phenotypically different from anti-AChR antibody-positive MG (AChR-MG) by prominent involvement of bulbar muscles and rapid progression to myasthenia crisis. Furthermore, they show a poor response to acetylcholinesterase inhibitors (ACEI), intravenous immunoglobulin (IVIg), standard immunosuppressant therapies, and thymectomy (5–8).

AChR-MG has been divided into distinct groups according to age at onset and the pathology of the thymus (9). Several studies have suggested the clinical differences among age subgroups, including distribution of muscle weakness, disease severity and response to immunotherapy (10–14). In MuSK-MG, it was reported that the majority were women aged between 30 and 40 years old (7, 15). However, recent data from our MG cohort indicated that the incidence of MuSK-MG in old patient has been increasing mainly due to the aging of the general population. MuSK-MG is still considered as a rare disease entity and no studies compared the clinical features among different age subgroups. We thus summarized the clinical features, longitudinal courses, and treatment outcome in a cohort of 69 MuSK-MG patients gathered from five tertiary referral centers in China and compared the difference among early, late and very-late-onset (16) subgroups.

## Methods

### Study Design and Patient Recruitment

**Figure 1:**
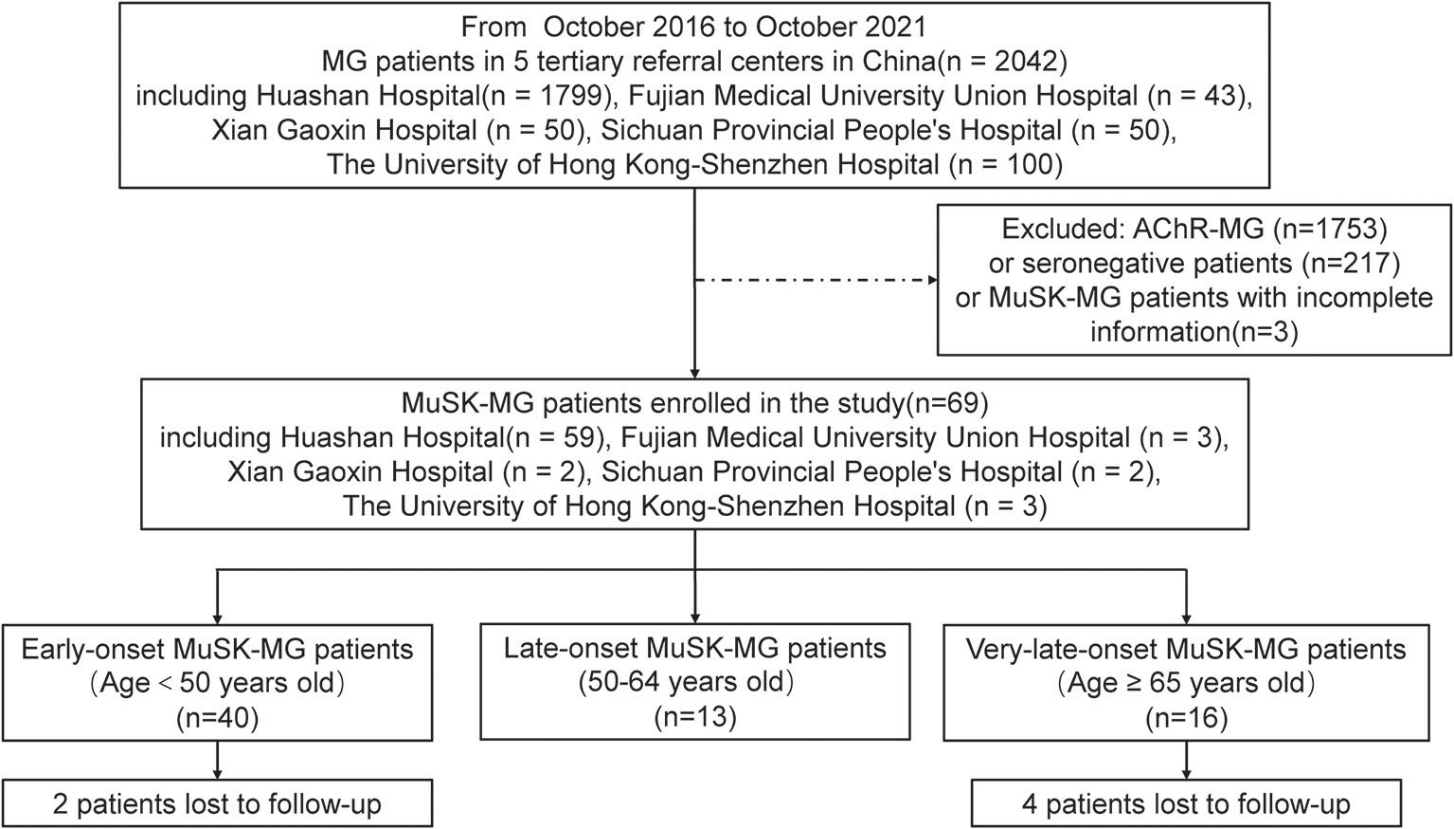
Flowchart for the process of patient inclusion.

In this observational retrospective multicenter study, we selected MuSK-MG patients in five tertiary referral centers from October 2016 to October 2021. These tertiary referral centers included Huashan Hospital, Fudan University in Shanghai, Fujian Medical University Union Hospital in Fujian Province, Xi'an Gaoxin Hospital in Shanxi Province, Sichuan Provincial People's Hospital in Sichuan Province, and the University of Hong Kong-Shenzhen Hospital in Guangdong Province (Figure 1). The onset age of all patients was older than 16 years. We classified the patients into the following subgroups: early-onset (patients with age at onset younger than 50 years old), late-onset (patients with age at onset 50–64 years old), and very-late-onset (patients with age at onset no ≥65 years old) subgroups. Written informed consent was granted by each patient and the study was approved by the Institutional Review Board of Huashan Hospital, Fudan University.

### Evaluation and Collection

We evaluated the following variables: demographic characteristics (gender, age at onset); diagnostic delay, defined as the time from the date of onset to diagnosis; Myasthenia Gravis Foundation of America (MGFA) classification (17) at disease onset and at maximal worsening; the time from onset to maximal worsening; distribution of muscle weakness at onset, 3 months, 6 months, and 1 year after onset, respectively; disease progression, defined as a new muscle group involvement 1 month after onset, including progression from ocular to bulbar muscles, from ocular to bulbar and limb muscles, from limb to bulbar muscles, from bulbar to limb muscles, and from any to respiratory muscles; myasthenic crisis (MC), defined as an event that requires mechanical ventilation because of severe involvement of respiratory muscles (18); the time from onset to MC; immunotherapy, including steroids, IVIg, plasma exchange (PE), conventional non-steroid immunosuppressant (azathioprine, tacrolimus, mycophenolate mofetil, cyclophosphamide, cyclosporine) and rituximab; refractory MuSK-MG, defined as MGFA postintervention status (MGFA-PIS) (19) unchanged or worse after steroids and at least one other non-steroid immunosuppressant; comorbid disease, including thyroid abnormalities, urticaria, eczema, hypertension, diabetes mellitus, etc.; minimal symptom expression (MSE), defined as the MG-Related Activities of Daily Living score (MG-ADL) is 0 or 1 score (20); follow-up period, defined as the time from disease onset to the last visit; the positive rate of repetitive nerve stimulation (RNS) at 3 Hz at the time of initial diagnosis.

### Statistical Analysis

Continuous variables that followed a normal distribution are presented as the mean ± standard deviation. Non-normally distributed data are presented as the median (interquartile range, IQR). Categorical variables are expressed as frequencies (percentages). Missing data were dropped as they were <20% of the sample for the relevant variables. Differences between subgroups were evaluated using the chi-square test and Fisher exact test for categorical variables, and the Kruskal Wallis H test to compare quantitative variables. *P*-values were adjusted by the Berforroni method. Kaplan–Meier curves and log-rank tests were used to compare the time and rate to achieve MSE status after rituximab treatment among three subgroups. A significant difference was defined as *p* < 0.05. Statistically significant variables were analyzed within each age group. Data analysis was carried out using IBM SPSS version 25.0 (SPSS Inc., Chicago, IL, USA). Diagram generation were all conducted in R version 3.63 (R Foundation for Statistical Computing, Vienna, Austria).

## Results

### Clinical Features of MuSK-MG Cohort

**Figure 2:**
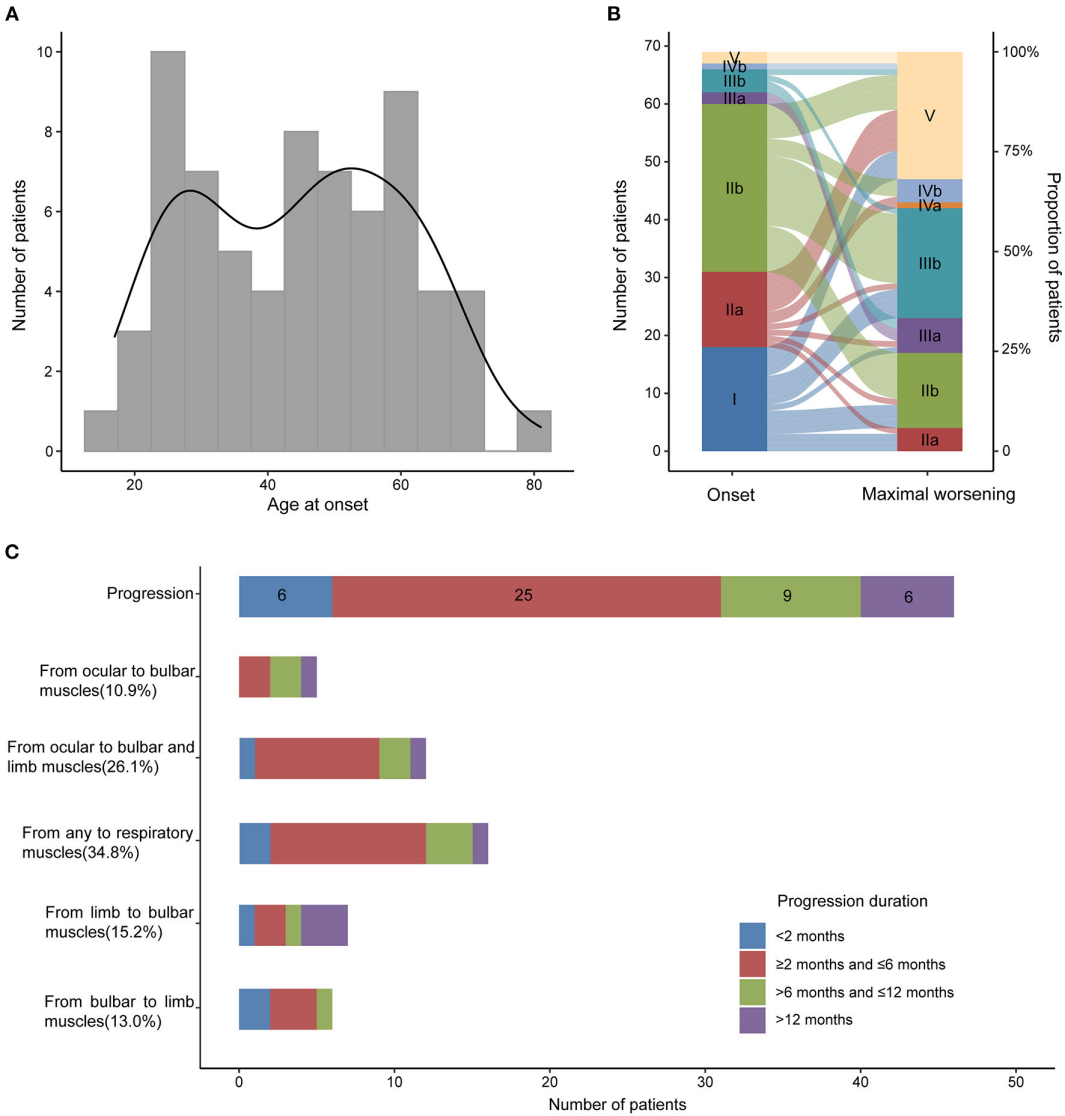
Age at onset, MGFA classification at the onset and maximal worsening and disease progression in MuSK-MG. **(A)** Age at onset of 69 MuSK-MG patients in our cohort; **(B)** MGFA classification at the onset and at maximal worsening during the period from disease onset to the last follow-up; **(C)** Muscle involvement and disease progression of all the patients.

In our MG cohort of 2,042 patients from five tertiary referral centers, about 3.5% (72) patients are MuSK-MG, comprising 24.9% (72/289) AChR-negative patients. We finally included 69 MuSK-MG in this study. Demographics and clinical features were summarized in Table 1. The patients showed a female predominance (55/69), and the mean age at onset was 44.70 ± 15.84 years old, with a broad range of 17–81 years old (Figure 2). The median diagnostic delay was 5 [(IQR) 1–8.5] months and the median disease course was 34 [(IQR) 16.5–56] months. At disease onset, most patients (29/69) were classified as MGFA IIb (Figure 2) and the frequencies of bulbar, limb, and extraocular muscle involvement were 53.6, 29.0, and 69.6%, respectively. Fluctuating weakness was reported in 69.6% (48/69) patients and 80.4% (41/51) showed a positive neostigmine test. Regarding electrophysiological examination, 63 patients underwent repetitive nerve stimulation (RNS) test and 71.4% showed an abnormal decrease at low-frequency stimulation (3 Hz) and the muscle with the highest sensitivity was orbicularis oculi (53.6%). Abd Pollicis Brevis, frontalis, deltoid and trapezius showed a relatively low positive rate of 12.5, 16.1, 20, and 21.8%, respectively (Supplementary Table 1). Nineteen patients combined with other chronic diseases, including eight with hypertension, six with diabetes mellitus, five with hyperlipidemia, five with hepatitis B, two with latent tuberculosis, and one with breast cancer but no checkpoint inhibitor usage. Coexisted other autoimmune diseases were reported in 18 patients, including eight with thyroid abnormalities, three with urticaria, one with eczema, and eleven with positive antinuclear-antibody (ANA).

**Table 1:** Clinical features of early-onset, late-onset, and very-late-onset MuSK-myasthenia gravis (MuSK-MG).

**Variables**	**Total** *N* **= 69**	**Early-onset** *N* **= 40**	**Late-onset** *N* **= 13**	**Very-late-onset** *N* **= 16**	***P*** **-value**
Female: male	55:14	34:6	9:4	12:4	0.384
Age at onset (years old) (mean ± SD)	44.70 ± 15.84	33.43 ± 9.49	53.85 ± 2.34	65.44 ± 5.37	***0.000***
Disease course (m) [median (IQR)]	34 (16.5–56)	34.5 (17.25–63.25)	48 (27–90.5)	18 (14.25–31.5)	**0.029^c^**
Diagnostic delay (m) [median (IQR)]	5 (1–8.5)	5 (1.25–8.75)	5 (2–13.5)	4 (1–6)	0.526
Positive fatigue test, *n* (%)	57/64 (89.1%)	31/35 (88.6%)	12/13 (92.3%)	14/16 (87.5%)	1^*^
Positive neostigmine test, *n* (%)	41/51 (80.4%)	19/27 (70.4%)	10/11 (90.9%)	12/13 (92.3%)	0.230^*^
Fluctuating weakness, *n* (%)	48 (69.6%)	27 (67.5%)	11 (84.6%)	10 (62.5%)	0.427^*^
RNS test positive, *n* (%)	45/63 (71.4%)	26/34 (76.5%)	11/13 (84.6%)	8/16 (50.0%)	0.090^*^
MGFA classification at onset					0.644^*^
I, *n* (%)	18 (26.1%)	11 (27.5%)	4 (30.8%)	3 (18.8%)	
II, *n* (%)	42 (60.9%)	23 (57.5%)	9 (69.2%)	10 (62.5%)	
III, *n* (%)	6 (8.7%)	5 (12.5%)	0	1 (6.3%)	
IV, *n* (%)	1 (1.4%)	0	0	1 (6.3%)	
V, *n* (%)	2 (2.9%)	1 (2.5%)	0	1 (6.3%)	
MGFA classification at maximal worsening					0.321^*^
II, *n* (%)	17 (24.6%)	10 (25.0%)	4 (30.8%)	3 (18.8%)	
III, *n* (%)	25 (36.2%)	15 (37.5%)	4 (30.8%)	6 (37.5%)	
IV, *n* (%)	5 (7.2%)	2 (5.0%)	3 (23.1%)	0	
V, *n* (%)	22 (31.9%)	13 (32.5%)	2 (15.4%)	7 (43.8%)	
**Comorbid disease**					
Hypertension, *n* (%)	8 (11.6%)	1 (2.5%)	2 (15.4%)	5 (31.3%)	0.006^b^, ^*^
Diabetes mellitus, *n* (%)	6 (8.7%)	0	1 (7.7%)	5 (31.3%)	0.001^b^, ^*^
Hyperlipidemia, *n* (%)	5 (7.2%)	0	2 (15.4%)	3 (18.8%)	0.013^a^, ^b^, ^*^
Hepatitis B, *n* (%)	5 (7.2%)	4 (10.0%)	1 (7.7%)	0	0.579^*^
Latent tuberculosis, *n* (%)	2 (2.9%)	1 (2.5%)	0	1 (6.3%)	0.668^*^
Tumor, *n* (%)	1 (1.4%)	0	0	1 (6.3%)	0.420^*^
**Other autoimmune disease**					
Thyroid abnormalities, *n* (%)	8 (11.6%)	2 (5.0%)	0	6 (37.5%)	0.003^b^, ^*^
Urticaria, *n* (%)	3 (4.3%)	2 (5%)	1 (7.7%)	0	0.762^*^
Eczema, *n* (%)	1 (1.4%)	1 (7.7%)	0	0	0.188^*^
Positive ANA, *n* (%)	11/41 (26.8%)	4/24 (16.7%)	2/6 (33.3%)	5/11 (45.5%)	**0.175** ^*^

a *Early-onset vs. late-onset*.

b *Early-onset vs. very-late-onset*.

c *Late-onset vs. very-late-onset*.

* *Using Fisher exact test. The bold and italic values mean significant differences*.

*RNS, repetitive nerve stimulation; MGFA, Myasthenia Gravis Foundation of America; ANA, antinuclear-antibody*.

**Figure 3:**
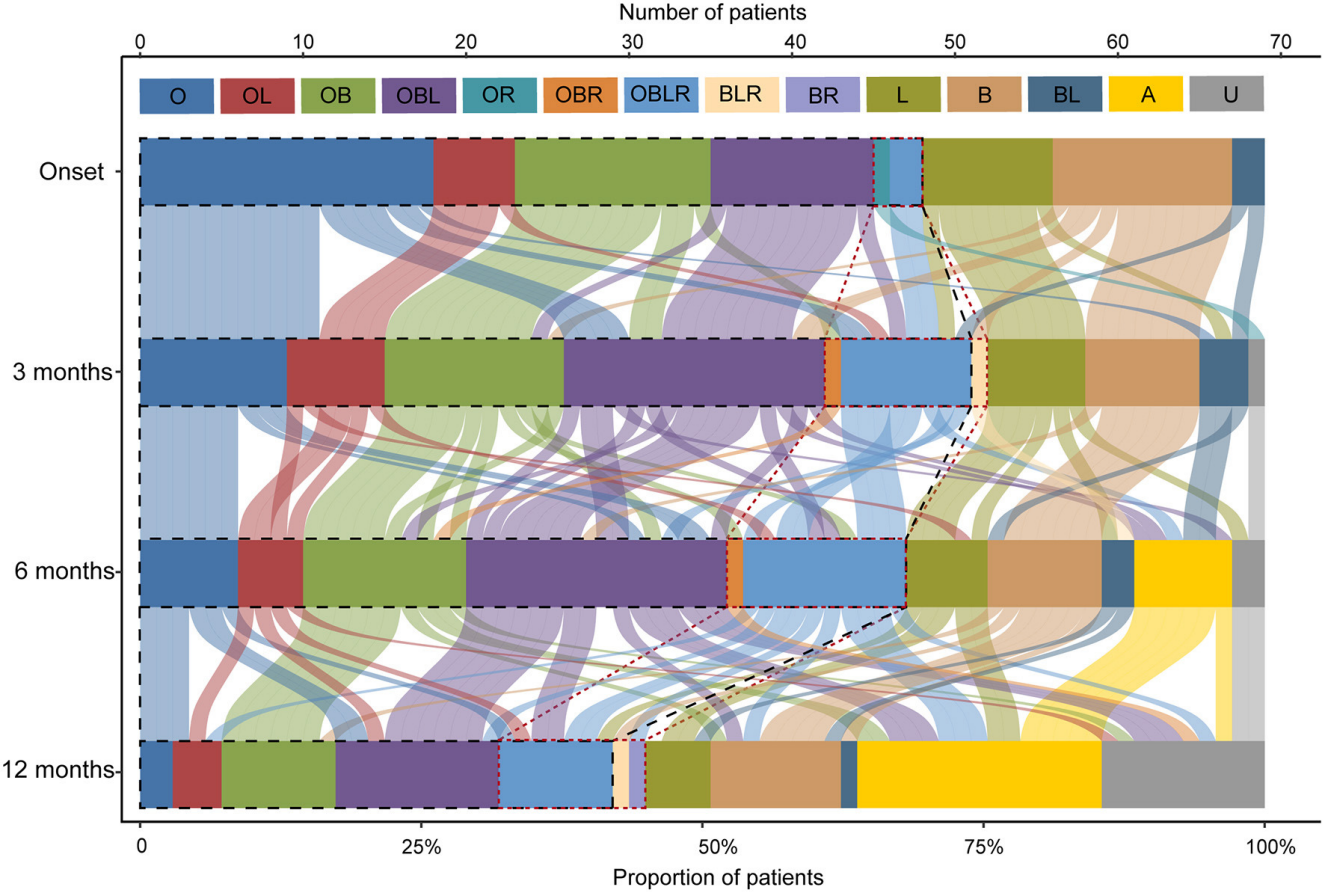
Point-in-time weakness distribution and shifts over time. The point-in-time weakness distribution at disease onset and 3, 6, 12months after onset. O, extraocular muscles; B, bulbar muscles; L, limb or neck muscles; R, respiratory muscles; and their multiple combinations. A, asymptomatic; U, unknown, patients who were lost to follow-up at this time point. The black dotted line outlined the patients with ocular involvement and the red dotted line outlined the patients with respiratory involvement.

Forty-six out of 69 patients displayed disease progression, most of which (31/466) occurred in the first 6 months (Figure 2). Myasthenic crisis (MC) occurred in 31.9% (22/69) patients, and 50% (11/22) showed MC within 6 months after onset. The median duration from onset to disease progression was 4.5 [(IQR) 2–9.25] months and from onset to MC was 7 [(IQR) 2.75–13] months. Longitudinal disease progression and weakness distribution were shown in Figure 3 and Table 2. Eighteen out of 69 patients showed a pure extraocular and 11/69 showed a pure bulbar phenotype at the onset. Two (2/18) patients remained pure extraocular involvement 1 year after onset and the two patients progressed to generalized MG 15 months (bulbar and limb involvement) and 48 months (bulbar involvement) after onset, respectively. Four patients remained pure bulbar involvement 1 year after onset and no further progression was observed. The proportion of respiratory involvement (11/67) was the highest 6 months after onset and was decreased to 9/59 6 months later, perhaps due to the immunotherapy.

**Table 2:** Weakness distribution and disease progression in early-onset, late-onset, and very-late-onset MuSK-myasthenia gravis.

**Variables**	**Total** *N* **= 69**	**Early-onset** *N* **= 40**	**Late-onset** *N* **= 13**	**Very-late-onset** *N* **= 16**	***P*** **-value**
**Weakness distribution at onset**					
Extraocular, *n* (%)	48 (69.6%)	27 (67.5%)	10 (76.9%)	11 (68.8%)	0.877^*^
Bulbar, *n* (%)	37 (53.6%)	22 (55%)	7 (53.8%)	8 (50%)	0.945^*^
Limbs, *n* (%)	20 (29.0%)	9 (22.5%)	3 (23.1%)	8 (50%)	0.125^*^
Neck, *n* (%)	20 (29%)	11 (27.5%)	4 (30.8%)	5 (31.3%)	0.937^*^
Respiratory, *n* (%)	3 (4.3%)	1 (2.5%)	0	2 (12.5%)	0.221^*^
**Weakness distribution 3 months after onset**					
Extraocular, *n* (%)	51/68 (75%)	29 (72.5%)	10 (76.9%)	12/15 (80%)	0.926^*^
Bulbar, *n* (%)	47/68 (69.1%)	27 (67.5%)	8 (61.5%)	12/15 (80%)	0.543^*^
Limbs, *n* (%)	33/68 (48.5%)	16 (40%)	5 (38.5%)	12/15 (80%)	0.022^b^,^*^
Neck, *n* (%)	29/68 (42.6%)	18 (45%)	5 (38.5%)	6/15 (40%)	0.891^*^
Respiratory, *n* (%)	10/68 (14.7%)	5 (12.5%)	0	5/15 (33.3%)	0.038^*^
**Weakness distribution 6 months after onset**					
Extraocular, *n* (%)	47/67 (70.1%)	27/39 (69.2%)	8 (61.5%)	12/15 (80%)	0.579^*^
Bulbar, *n* (%)	47/67 (70.1%)	26/39 (66.7%)	7 (53.8%)	14/15 (93.3%)	0.044^c^,^*^
Limbs, *n* (%)	28/67 (41.8%)	12/39 (30.8%)	3 (23.1%)	13/15 (86.7%)	0.000^b^,^c^,^*^
Neck, *n* (%)	22/67 (32.8%)	11/39 (28.2%)	3 (23.1%)	8/15 (53.3%)	0.178^*^
Respiratory, *n* (%)	11/67 (16.4%)	5/39 (12.8%)	0	6/15 (40%)	0.013^c^,^*^
**Weakness distribution 1 year after onset**					
Extraocular, *n* (%)	29/59 (49.2%)	18/35 (51.4%)	6/12 (50%)	5/12 (41.7%)	0.934^*^
Bulbar, *n* (%)	35/59 (59.3%)	21/35 (60%)	8/12 (66.7%)	6/12 (50%)	0.715^*^
Limbs, *n* (%)	19/59 (32.2%)	10/35 (28.6%)	3/12 (25%)	6/12 (50%)	0.375^*^
Neck, *n* (%)	17/59 (28.8%)	9/35 (25.7%)	3/12 (25%)	5/12 (41.7%)	0.623^*^
Respiratory, *n* (%)	9/59 (15.3%)	2/35 (5.7%)	3/12 (25%)	4/12 (33.3%)	0.036^b^,^*^
Time from onset to maximal worsening (m), [median (IQR)]	4 (1–11.5)	4 (1–14)	7 (3.5–13)	2.5 (0.475–6)	0.288
Progress, *n* (%)	46 (66.7%)	26 (65%)	8 (61.5%)	12 (75%)	0.778^*^
Progression ≤ 6 months from onset, *n* (%)	31 (44.9%)	18 (45%)	3 (23.1%)	10 (62.5%)	0.105^*^
Time from onset to progression (m), median (IQR)	4.5 (2–9.25)	4 (2.75–7.5)	9.5 (3.25–39.5)	3.5 (2–6)	0.097
Myasthenic crisis, *n* (%)	22 (31.9%)	13 (32.5%)	2 (15.4%)	7 (43.8%)	0.271^*^
Myasthenic crisis ≤ 6 months from onset, *n* (%)	11 (15.9%)	6 (15%)	0	5 (31.3%)	0.088^*^
Time from onset to crisis (m) [median (IQR)]	7 (2.75–13)	8 (3.5–16.5)	10.5 (9-)	3 (1–11)	0.417

b *Early-onset vs. very-late-onset*.

c *Late-onset vs. very-late-onset*.

* *Using Fisher exact test. The bold and italic values mean significant differences*.

Table 3 showed details of the treatment and prognosis of the patients. All patients were followed up with the median follow-up period of 32 [(IQR) 13.5–56)] months. Sixty-four (94.1%) patients received steroids, 25 (39.1%) received at least one non-steroid immunosuppressant, 46 (66.7%) received rituximab and 39 (60.9%) reached MSE status. Among the patients who received rituximab, 44 patients were administered 600 mg rituximab every 6 months and two patients from Xi'an Gaoxin Hospital used a regimen of 4 weekly infusions of 100 mg followed by maintenance therapy depending on the emergence of CD20^+^B-cells. Sixteen patients (23.5%) were refractory MuSK-MG and 13/16 patients received rituximab and 8/13 reached MSE. Thirteen patients (19.1%) attained MSE status using conventional treatments. Twenty-six patients (38.2%) did not reach MSE status until the use of rituximab. Among 41 patients who did not reach the status of MSE before administrating RTX with 600 mg regimen, although no significant difference (log-rank test: *p* = 0.075), a trend that more patients in early course of disease (≤ 1 year) reached the status of MSE was observed (Supplementary Figure 1). One patient underwent thymectomy and the histopathologic diagnosis was thymic hyperplasia.

**Table 3:** Treatment and prognosis in early-onset, late-onset, and very-late-onset MuSK-myasthenia gravis (MuSK-MG).

**Variables**	**Total** *N* **= 69**	**Early-onset** *N* **= 40**	**Late-onset** *N* **= 13**	**Very-late-onset** *N* **= 16**	***P*** **-value**
**Treatment**					
PE, *n/N* (%)	27/68 (39.7%)	16/39 (41%)	3/13 (23.1%)	8/16 (50%)	0.325^*^
IVIg, *n/N* (%)	30/68 (44.1%)	19/39 (48.7%)	5/13 (38.5%)	6/16 (37.5%)	0.709^*^
ACEI, *n/N* (%)	64/67 (95.5%)	37/38 (97.4%)	12/13 (92.3%)	15/16 (93.8%)	0.398^*^
Steroid, *n/N* (%)	64/68 (94.1%)	37/39 (94.9%)	12/13 (92.3%)	15/16 (93.8%)	1^*^
Rituximab, *n/N* (%)	46/68 (66.7%)	20/39 (75.0%)	7/13 (53.8%)	9/16 (56.3%)	0.202^*^
MMF, *n/N* (%)	5/68 (7.4%)	1/39 (2.6%)	2/13 (15.4%)	2/16 (12.5%)	0.121^*^
Tacrolimus, *n/N* (%)	12/68 (17.6%)	7/39 (17.9%)	3/13 (23.1%)	2/16 (12.5%)	0.829^*^
AZA, *n/N* (%)	12/68 (17.6%)	8/39 (20.5%)	2/13 (15.4%)	2/16 (12.5%)	0.908^*^
CTX, *n*/*N* (%)	1/68 (1.5%)	0	1/13 (7.7%)	0	0.191^*^
Cyclosporine, *n/N* (%)	2/68 (2.9%)	0	1/13 (7.7%)	1/16 (6.3%)	0.178^*^
Thymectomy, *n/N* (%)	1/68 (1.5%)	1/39 (2.6%)	0	0	1^*^
Refractory, *n/N* (%)	16/68 (23.5%)	10/39 (25.6%)	3/13 (23.1%)	3/16 (18.8%)	0.925^*^
Rituximab, *n/N* (%)	13/16 (81.3%)	9/10 (90%)	3/3 (100%)	1/3 (33.3%)	0.143^*^
Median follow-up period (m), median (IQR)	32 (13.5–56)	33.5 (15.5–63.25)	48 (27–90.5)	15 (9.75–20.75)	***0.007^a^,^b^***
MSE, *n/N* (%)	39/64 (60.9%)	25/38 (65.8%)	9/13 (69.2%)	5/13 (38.5%)	0.218^*^
Time from onset to MSE (m), median (IQR)	11 (7.0–28.0)	10 (7.0–27.5)	15 (12.0–49.5)	10 (8.5–23.5)	0.254
Time from onset to receiving rituximab (m), median (IQR)	9 (6.0–24.75)	10 (5.75–49.5)	18 (12–65)	6 (5.5–7.5)	***0.039^b^***

a *Early-onset vs. very-late-onset*.

b *Late-onset vs. very-late-onset*.

* *Using Fisher exact test. The bold and italic values mean significant differences*.

*PE, plasma exchange; IVIg, intravenous immunoglobulin; ACEI, acetylcholinesterase inhibitors; MMF, mycophenolate mofetil; AZA, azathioprine; CTX, cyclophosphamide; MSE, minimal symptom expression*.

### Difference Among Age Subgroups in MuSK-MG

According to age at onset, 40 patients (58%) were subclassified into early-onset, 13 (18.8%) into late-onset and 16 (23.2%) into very-late-onset subgroup. Clinical features of each subgroup were summarized in Tables 1–3. All subgroups were female-dominant and no difference of diagnostic delay was found. Among three subgroups, the positive rate of fatigue test and neostigmine test, the complaint of weakness fluctuating showed no difference, either. As for combined diseases, hypertension (5/16 vs. 1/40, *p* = 0.006), diabetes mellitus (5/16 vs. 0/40, *p* = 0.001) and hyperlipidemia (3/16 vs. 0/40, *p* = 0.013) occurred more frequently in very-late-onset subgroup than in early-onset subgroup. More patients in very-late-onset subgroup showed thyroid abnormalities (6/16 vs. 2/40, *p* = 0.003).

At disease onset, no differences were observed regarding MGFA classification and weakness distribution among three subgroups. Compared patients with early-onset, patients with very-late-onset onset showed a higher frequency of limb involvement (12/15 vs.16/40, respectively, *p* = 0.022) 3 months after onset. Six months after onset, more patients in very-late-onset subgroup had bulbar and respiratory involvement than that in late-onset subgroup (bulbar: 14/15 vs. 26/39, *p* = 0.044; respiratory: 6/15 vs. 0/13, *p* = 0.013). In addition, more patients in very-late-onset subgroup showed weakness of limbs (86.7%, *p* < 0.001) than that in the other two subgroups. One year after onset, a higher frequency of respiratory involvement was reported in very-late-onset than in early-onset subgroups (4/12 vs. 2/35, *p* = 0.036) (Table 2). The time from onset to progression, the time from onset to maximal worsening, or from onset to MC among subgroups were not statistically different (*p* > 0.05).

**Figure 4:**
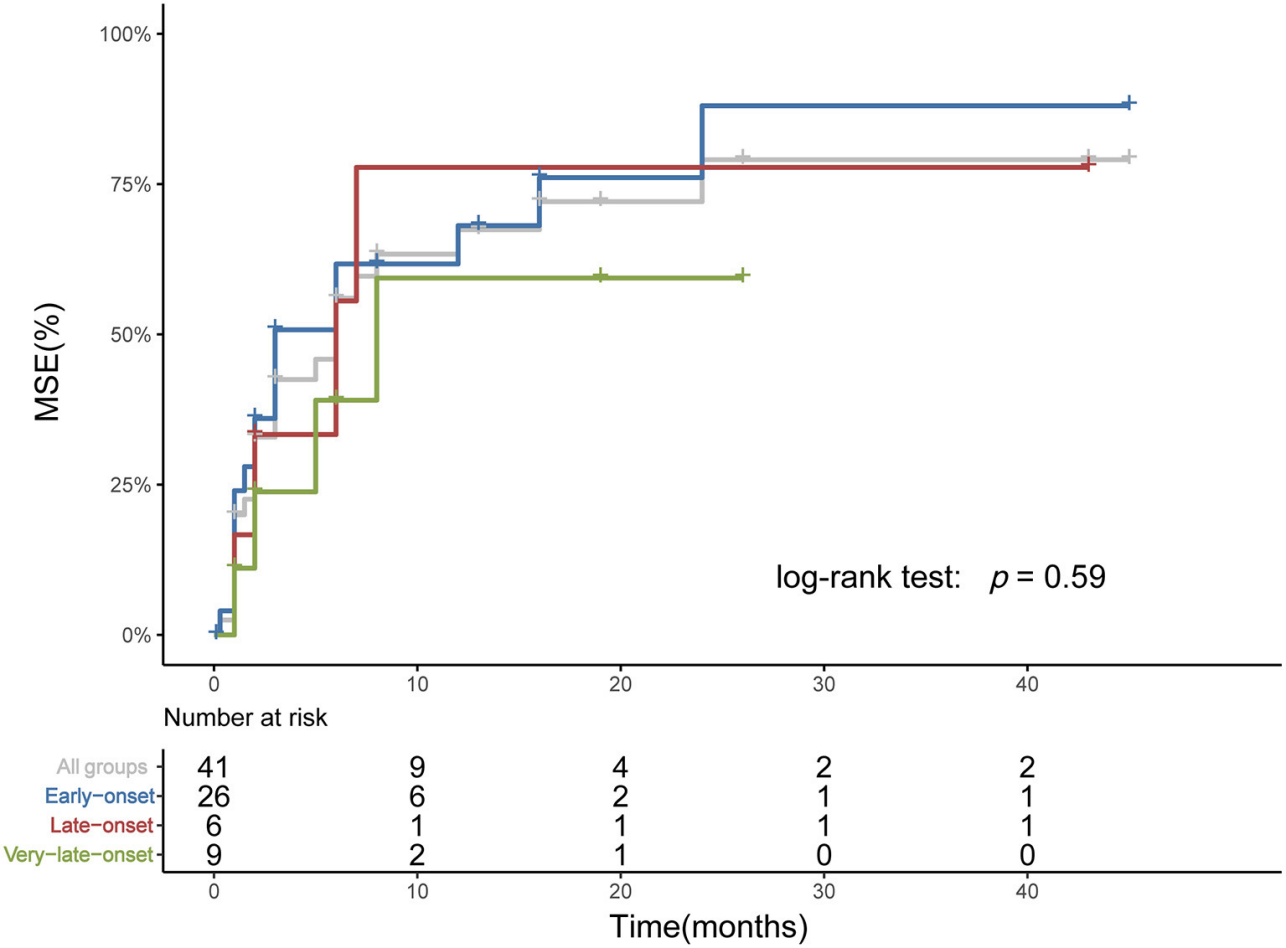
Time from rituximab treatment to achieving MSE among subgroups. The Kaplan-Meier plot showed the time from rituximab treatment to reaching MSE. Three patients who had achieved MSE before rituximab and two patients from Xian Gaoxin Hospital with distinct regimen were not included. No significant difference was identified among subgroups.

Among age subgroups, the number of patients treated with ACEI, glucocorticoid, rituximab, PE, and IVIg was not significantly different. The proportion of refractory MuSK-MG did not differ from each other, either. The rate of patients who had reached MSE status (25/38, 9/13, 5/13, respectively, *p* > 0.05) and the time from onset to MSE status showed no significant difference (Table 3). For patients treated with rituximab, a shorter time from onset to receiving rituximab was found in very-late-onset subgroup compared to late-onset subgroup {6 [(IQR) 5.5–7.5] months vs. 18 [(IQR) 12–65] months, *p* = 0.039}. No significant difference in the rate to achieve MSE and the time from rituximab treatment to achieving MSE was identified among three subgroups (*p* > 0.05) (Figure 4).

## Discussion

As reported in previous studies, patients with MuSK-MG in our cohort also showed predominant involvement of extraocular, bulbar and respiratory muscles (7). However, the age at onset showed a bimodal age pattern of incidence, with one peak in individuals younger than 40 years, one peak in individuals aged 40–70 years old, which was different from the conclusion that the age at onset was rarely after 60 (7, 21, 22). The increase in the incidence of very-late-onset MuSK-MG might be a result of the aging of general population and expansion of life expectancy. The acknowledgment of clinical features of MuSK-MG and the increase in sensitivity and specificity of diagnostic methods also lead to an increase of diagnostic yield and a decline of misdiagnosis (19, 23, 24). It can also be attributable to the changes in the immune system during aging, including the increase in inflammatory reactions and the higher production of autoantibodies (25).

Growing evidence from clinical researches suggested the differences in clinical profile, natural history and treatment outcome among age subgroups in AChR-MG. Female cases outnumber male cases by three to one in early-onset patients (9). They were more likely to present with an initially generalized disease and a high level of anti-AChR antibodies associated with thymic follicular hyperplasia (10, 26). Late and very-late-onset AChR-MG was more common in males and more frequently had seropositive acetylcholine receptor antibodies and ocular MG (10, 11, 14, 27). The therapeutic management of these two groups is more complex because of comorbidities (28). But Cortés-Vicente et al. found although very-late-onset patients had a higher frequency of life-threatening events, their long-term outcomes were good, with less requirement for immunosuppressive medications and a lower probability of being refractory (10).

However, the difference of clinical features, longitudinal disease progression and treatment outcomes among age subgroups in MuSK-MG is not clear. In our cohort, patients in very-late-onset subgroups showed a higher proportion of combined chronic diseases, including hypertriton, diabetes, thyroid abnormalities, etc. There was no difference of MGFA classification and weakness distribution at disease onset, but patients in the very-late-onset subgroup showed an early involvement of limb, bulbar and respiratory muscles in the disease course, especially in the first 6 months. As a result, very-late-onset patients started rituximab treatment earlier. Concerning the treatment outcome, three subgroups attained similar outcomes with no significant difference in the rate and time of remission.

We found that the distinction among age subgroups in MuSK-MG was not as great as that in AChR MG, which might be due to distinct pathogenesis between AChR-MG and MuSK-MG: (1) There are functional and morphological abnormalities of the thymus in the pathogenesis of AChR-MG. B-cell infiltrations are associated with thymic hyperplasia of lymphoproliferative, which could be identified in more than 80% early-onset patients (26, 29). The thymus of late-onset patients usually shows normal-for-age atrophy. Although the mechanisms are not understood, the presence of anti-striational and anti-cytokine autoantibodies in late-onset patients strongly suggests similar role with thymoma (30, 31). In contrast, thymic hyperplasia and thymoma are rarely observed in MuSK-MG. (2) AChR antibodies are mainly of IgG1 and IgG3 subtypes, which can bind to C1q to activate the complement cascade. The number of anti-AChR antibody producer, including plasma cells and memory B cells, decreased in the elderly (32, 33). By comparison, MuSK-MG autoantibodies are mainly of the IgG4 subclass, which undergo Fab-arm exchange as a prerequisite for pathogenic capacity (34). They are produced by plasmablasts, which are found in similar proportions in all age subgroups (35). (3) Anti-AChR antibodies modulate myogenic markers and lead to impaired muscle regeneration, while the effect of anti-MuSK antibodies on regeneration remains unclear (36). It is noteworthy that satellite cells are quantitatively and functionally age-dependent, with a marked decline with age (37, 38), this might explain the rapid progression in patients with very-late-onset MuSK-MG.

This study has several limitations. First, this was a retrospective study, and therefore, potential selection bias, missing data bias and recall misclassification could not be avoided. Second, only MSE was used to evaluate the prognosis of MG, other prognostic outcomes such as the reduction in daily dosage of prednisone and the maintenance of asymptomatic were not analyzed. Third, the sample size of the cohort, especially in late-onset and very-late-onset subgroups, is still small. To better understand the distinction of clinical features, longitudinal disease progression and treatment outcome in MuSK-MG among age subgroups, further prospective studies with larger sample size are required.

In conclusion, our results are consistent with previous studies, which showed MuSK-MG patients usually manifested as acute onset and predominant bulbar and respiratory involvement with female dominance. Compared with late-onset patients, very-late-onset patients displayed an early involvement of limb, bulbar and respiratory muscles in the disease course, which might prompt their earlier usage of rituximab. The majority MuSK-MG patients can benefit from rituximab treatment regardless of age at onset.

## Data Availability Statement

The original contributions presented in the study are included in the article/Supplementary Material, further inquiries can be directed to the corresponding author/s.

## Ethics Statement

The studies involving human participants were reviewed and approved by the Institutional Review Board of Huashan Hospital, Fudan University. The patients/participants provided their written informed consent to participate in this study.

## Author Contributions

JX and WZ designed the study and revised the manuscript. CZ supervised the study. YZ and JC collected all the data and drafted the manuscript. CY conducted statistical analysis. ZL, ST, LZ, SL, JS, XH, and YW collected clinical and laboratory data. All authors contributed to the article and approved the submitted version.

## Funding

This research was supported by the National Natural Science Foundation of China (No. 81901279).

## Conflict of Interest

The authors declare that the research was conducted in the absence of any commercial or financial relationships that could be construed as a potential conflict of interest.

## Publisher's Note

All claims expressed in this article are solely those of the authors and do not necessarily represent those of their affiliated organizations, or those of the publisher, the editors and the reviewers. Any product that may be evaluated in this article, or claim that may be made by its manufacturer, is not guaranteed or endorsed by the publisher.

## Abbreviations

Ab: Autoantibody
ACEI: Acetylcholinesterase inhibitors
AChR: Acetylcholine receptor
ANA: Antinuclear-antibody
IQR: Interquartile range
IVIg: Intravenous immunoglobulin
MC: Myasthenic crisis
MG: Myasthenia Gravis
MG-ADL: MG-Related Activities of Daily Living score
MGFA: Myasthenia Gravis Foundation of America
MGFA-PIS: MGFA postintervention status
MSE: Minimal symptom expression
MuSK: Muscle specific tyrosine kinase
PE: Plasma exchange
RNS: Repetitive nerve stimulation.
