# Supplementary material for: Clinical Features of Myasthenia Gravis With Antibodies to MuSK Based on Age at Onset: A Multicenter Retrospective Study in China
Source: Front Neurol. 2022 Apr 8;13:879261. doi: 10.3389/fneur.2022.879261 (PMC9033288; doi:10.3389/fneur.2022.879261)
Supplement: Supplementary file 1 [file Table_1.DOCX]

**Supplementary Table Electrophysiological features in early-onset, late-onset, and very-last-onset MuSK-myasthenia gravis (MG)**

| **Variables** | **Total**  **N=69** | **Early-onset**  **N=40** | **Late-onset**  **N=13** | **Very-late-onset**  **N=16** | **P value** |
| --- | --- | --- | --- | --- | --- |
| Positive RNS test, n/N (%) | 45/63(71.4%) | 26/34(76.5%) | 11/13(84.6%) | 8/16(50%) | 0.09* |
| Frontalis, n/N (%) | 9/56(16.1%) | 5/28(17.9%) | 2/12(16.7%) | 2/16(12.5%) | 1* |
| Nasalis, n/N (%) | 16/56(28.6%) | 8/28(28.6%) | 5/12(41.7%) | 3/16(18.8%) | 0.396* |
| Orbicularis oculi - eyelid, n/N (%) | 24/56(42.9%) | 12/28(42.9%) | 8/12(66.7%) | 4/16(25%) | 0.09* |
| Orbicularis oculi - orbit, n/N (%) | 22/56(39.3%) | 11/28(39.3%) | 5/12(41.7%) | 6/16(37.5%) | 1* |
| Deltoid, n/N (%) | 11/55(20%) | 7/28(25%) | 3/11(27.3%) | 1/16(6.3%) | 0.263* |
| Trapezius, n/N (%) | 12/55(21.8%) | 5/27(18.5%) | 4/12(33.3%) | 3/16(18.8%) | 0.581* |
| Abd Pollicis Brevis,n/N (%) | 7/56(12.5%) | 4/28(14.3%) | 1/12(8.3%) | 2/16(12.5%) | 1* |

^a^ early-onset vs. late-onset; ^b^ early-onset vs. very-late-onset; ^c^ late-onset vs. very-late-onset; ^*^ using Fisher exact test.

Abbreviations: RNS: repetitive nerve stimulation.


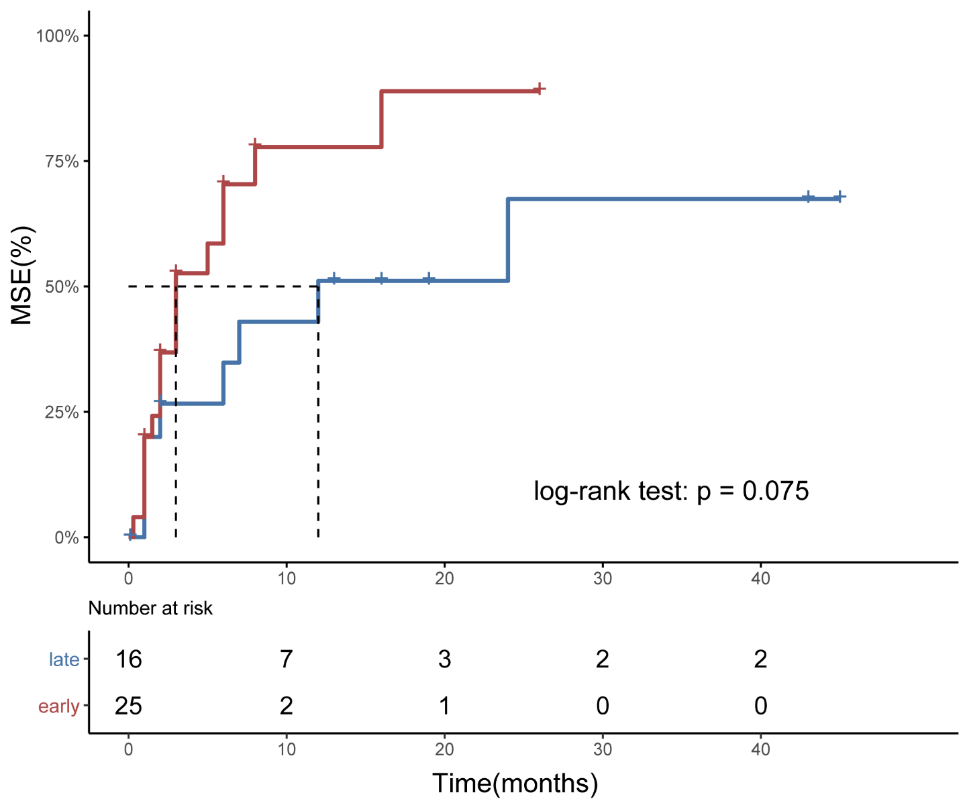


**Supplementary Figure** Time from rituximab treatment to achieving MSE between patients in early (≤1 year) and late (>1 year) courses of the disease.
